# Mesenchymal stem cell application in pulmonary disease treatment with emphasis on their interaction with lung-resident immune cells

**DOI:** 10.3389/fimmu.2024.1469696

**Published:** 2024-11-08

**Authors:** Ali Hazrati, Seyed Mohamad Javad Mirarefin, Kosar Malekpour, Arezou Rahimi, Arezou Khosrojerdi, Ashkan Rasouli, Susan Akrami, Sara Soudi

**Affiliations:** ^1^ Department of Immunology, School of Medicine, Tehran University of Medical Sciences, Tehran, Iran; ^2^ Department of Immunology, Faculty of Medical Sciences, Tarbiat Modares University, Tehran, Iran; ^3^ Department of Immunology, School of Medicine, Iran University of Medical Sciences, Tehran, Iran; ^4^ Infectious Diseases Research Center, Birjand University of Medical Sciences, Birjand, Iran; ^5^ Department of Immunology, School of Medicine, Shahid Beheshti University of Medical Sciences, Tehran, Iran; ^6^ Department of Microbiology, School of Medicine, Tehran University of Medical Sciences, Tehran, Iran

**Keywords:** pulmonary disease, inflammation, mesenchymal stem cells, exosomes, immune cells, immunomodulation

## Abstract

Due to the vital importance of the lungs, lung-related diseases and their control are very important. Severe inflammatory responses mediated by immune cells were among the leading causes of lung tissue pathology and damage during the COVID-19 pandemic. In addition, uncontrolled immune cell responses can lead to lung tissue damage in other infectious and non-infectious diseases. It is essential to control immune responses in a way that leads to homeostasis. Immunosuppressive drugs only suppress inflammatory responses and do not affect the homeostasis of reactions. The therapeutic application of mesenchymal stem cells (MSCs), in addition to restoring immune homeostasis, can promote the regeneration of lung tissue through the production of growth factors and differentiation into lung-related cells. However, the communication between MSCs and immune cells after treatment of pulmonary diseases is essential, and investigating this can help develop a clinical perspective. Different studies in the clinical phase showed that MSCs can reverse fibrosis, increase regeneration, promote airway remodeling, and reduce damage to lung tissue. The proliferation and differentiation potential of MSCs is one of the mechanisms of their therapeutic effects. Furthermore, they can secrete exosomes that affect the function of lung cells and immune cells and change their function. Another important mechanism is that MSCs reduce harmful inflammatory responses through communication with innate and adaptive immune cells, which leads to a shift of the immune system toward regulatory and hemostatic responses.

## 1 Introduction

As one of the vital organs for a healthy life, the lungs and their proper functioning are very important. The lungs directly interact with the external environment, and their functions can be affected by several inflammatory reactions caused by allergens, inflammatory mediators, and pathogens (1, 2). Different diseases may damage the lungs and disrupt some of their functions (3). The origins of these diseases can differ, and bacteria, viruses, genetics, lifestyle, and even inflammatory diseases have been identified (4). In many cases, viral or bacterial infections can cause damage to the airways. However, in some cases, uncontrolled inflammatory reactions caused by immune cells may be the leading cause of the pathology of the disease (4, 5). In addition, allergic diseases, including asthma, also lead to lung tissue involvement and are considered to be an inappropriate response of the immune system and its complications (type 1 hypersensitivity) (6). If the inflammation caused by immune system cells is uncontrolled, it can eventually lead to pulmonary fibrosis (7), which is associated with the irreversible loss of lung tissue. Therefore, the role of immune system cells in developing lung disease and controlling their responses is vital for maintaining lung tissue integrity.

Macrophages (MQs), dendritic cells (DCs), CD4^+^ T cells, and CD8^+^ T cells are among the main immune cell populations that are residents of the lung tissue (8). However, the recruitment of other immune cells, including neutrophils, is also observed in many acute inflammatory lung conditions (9). These cells are essential for maintaining lung tissue homeostasis and preventing harmful infections. Immune cell ratio changes can be observed in diseases where inflammation is the leading cause of injury (10, 11). For example, the regulatory T cell (Treg)/Th17 axis is one of the essential immune axes that can cause inflammatory damage to different tissues by changing the ratio of T cells toward Th17 differentiation (12, 13). Additionally, other types of T cells, such as Th2 and Th9 cells, may play a role in some conditions, such as allergic responses and stimulating the proliferation of fibroblasts through the production of cytokines, including interleukin 9 (IL-9), in pulmonary fibrosis (14–16). The balance between the inflammatory M1 and anti-inflammatory M2 phenotypes of macrophages is also essential for maintaining lung health (17, 18). M1 cells can damage lung tissue and alveoli by producing cytokines and stimulating the responses of Th1 cells (19). In addition, M1 macrophages, dendritic cells, and Th1 cells activate CD8^+^ T cells and subsequently cause damage.

In general, controlling the balance between immune system cells and their functions is needed to maintain lung tissue integrity. As mentioned, different factors can disrupt this balance, and therapeutic approaches that can restore it may help prevent lung damage.

Various treatments are suggested in the case of lung disease. In the case of allergic diseases involving the lungs, the primary treatment is to avoid the allergen (20). Various treatments are used for asthma, including cromolyn, inhibitors of leukotrienes, anti-IgE antibodies (omalizumab), and others, including salbutamol (21, 22). The treatment of infectious diseases related to the lungs differs according to the origin of the infection, whether it is bacterial, viral, or fungal, and includes antibiotics and anti-inflammatory drugs (23). During the COVID-19 pandemic, a monoclonal antibody against IL-6 (tocilizumab), which inhibits the function of IL-6 as an inflammatory cytokine, was used in patients with severe conditions (24). Different types of corticosteroids, including dexamethasone (25), have also been prescribed in some cases, which shows the importance of controlling inflammation in lung-related diseases. Although chemical treatments and antibodies based on suppressing inflammatory responses have shown promising results, a set of different treatments should be combined to prevent unwanted reactions. It appears that using a method that can act in a multifaceted manner helps improve lung function and is more patient-friendly.

Mesenchymal stromal/stem cells (MSCs) have attracted the attention of researchers in the field of lung disease treatment due to their therapeutic properties (26). The immunomodulatory function of these cells has been confirmed. MSCs perform their immunomodulatory functions through different mechanisms that include the production of anti-inflammatory cytokines and growth factors and the involvement of immune checkpoints (27, 28). Additionally, these cells have antifibrotic effects and can reverse fibrosis in this tissue (29). In addition, alveolar differentiation in lung progenitor organoid cultures has been shown to occur *in vitro* (30). By transferring mitochondria to damaged cells, MSCs can protect them from death caused by oxidative stress, leading to a change in their metabolism (31). Another feature of MSCs that leads to their therapeutic use is their ability to differentiate into different types of cells, which can be used in many diseases, including liver, orthopedic, and neurodegenerative diseases (32, 33).

MSCs produce exosomes and lipid bilayer vesicles with a size of 50 to 150 nm at a high rate. As small-scale cells, exosomes have high therapeutic potential but do not have the disadvantages of cell therapy (34). These vesicles contain different substances; transferring them to the target cell changes their functions. In some cases, by engineering exosomes, their therapeutic potential can be increased (35). One of these methods is the loading of drugs into these vesicles, which is very useful in the treatment of cancer. However, due to the proven immunomodulatory properties of these exosomes, they can also be used to treat inflammatory diseases. Considering the vital role of inflammation in the pathogenesis of lung diseases, in this review, we will focus on the effects of the therapeutic use of MSCs and their exosomes on these diseases through their effects on immune system cells.

## 2 The role of immune cells in lung disease pathogenesis

Immune system cells play a vital role in preventing lung-related infections. The flawless functioning of all components, from epithelial cells to complement components, is required to avoid harmful infections. In families with diseases such as immunodeficiency (Chronic Granulomatous Disease and Severe Combined Immunodeficiency Syndrome), one of the main challenges is infections that involve the lungs and threaten the patient’s life. Therefore, accurate and complete functioning of immune system components is required for proper lung function and the prevention of infectious diseases.

In some cases, damage to various tissues can occur due to the excessive activity of various cells in the immune system. The type of disease varies depending on which parts of the immune system are hyperactive. In some cases, the function of CD8^+^ T cells leads to the destruction of lung tissue cells and is considered a part of tissue pathogenesis. Pulmonary fibrosis can also occur due to the excessive activity of M2 macrophages and the proliferation of fibroblasts. Therefore, understanding the mechanisms related to immune system-mediated pathogenesis is very important for designing treatment protocols.

### 2.1 Macrophages

MQs are essential in the defense against pathogens throughout the body, including in lung tissue, and are among the leading players in innate immunity (36). These cells express different pattern recognition receptors (PRRs), including Toll-like receptors (TLRs), cytosolic receptors for recognizing foreign DNA, and scavenger receptors on their surface. They also express other types of molecules involved in antibodies recognizing opsonized components, including Fc receptors (FCRs) and complement receptors (CRs) (37, 38).

During lung inflammation, a combination of growth factors, cytokines, and damage-associated molecular patterns (DAMPs) released from cells leads to MQ recruitment and impacts MQ plasticity (39, 40). By expressing major histocompatibility complex (MHC)-II, CD80, and CD86 molecules after phagocytizing pathogens, MQs can present peptides derived from pathogens by MHC-II which leads to the activation of T cells (41). After being activated, MQs produce inflammatory cytokines and chemokines by upregulating NF-κB transcription factor expression and translocation to the nucleus in a Notch1-dependent manner (42), which may lead to the targeted migration of inflammatory immune cells and play a role in the pathogenesis of lung damage (43). Acute respiratory distress syndrome (ARDS), allergic asthma, chronic obstructive pulmonary disease (COPD), acute lung injury (ALI), COVID-19, and idiopathic pulmonary fibrosis (IPF) are among the diseases in which they are significant (44–46).

The excessive differentiation of macrophages into either M1 or M2 phenotypes and the disruption of the balance between these cell populations (M1/M2 axis) may play a role in the pathogenesis of various diseases (17, 47). M1 cells can damage lung cells by producing ROS; inflammatory cytokines such as IL-1B, tumor necrosis factor-alpha (TNF-α), and IL-6; and MIP-2 and MCP-1 (CCL2) chemokines, contributing to tissue damage (44, 48). Additionally, an increase in the proportion of M2 cells can contribute to fibrosis by producing cytokines such as transforming growth factor-beta (TGF-β) and increased collagen deposition (49). Additionally, by suppressing immune responses by producing IL-4 and IL-10, MQs may increase the susceptibility of this organ to various types of infection (50). According to recent studies, M2 cells, based on their functions, can be categorized into three subpopulations: M2a, M2b, and M2c (51). M2a cells typically play a role in inflammation associated with allergic reactions. M2b cells have different functions and play a central role in fibrosis and tissue remodeling (52). M2c macrophages help with the homeostasis of immune system responses through their anti-inflammatory and immunomodulatory functions. Therefore, maintaining the hemostasis between the ratio of M1 and M2 macrophages is essential for preserving lung health and proper function.

However, a new mechanism that defines the inhibitory role of some types of macrophages in airway inflammation is related to the formation of macrophage extracellular traps (METs). It has been shown that MQs can prevent the inflammation caused by other immune cells by producing these traps through peptidyl arginine deiminase type 4 (PAD4) and removing ambient particulate matter (PM) (53).

### 2.2 DCs and neutrophils

Neutrophils, also known as polymorphonuclear cells (PMNs) due to their nucleus shape, are present in circulation and have a short lifespan of a few hours (54). During infection, endothelial cells and fibroblasts produce chemokines that guide PMNs from the bloodstream to the inflamed region. Neutrophils express various receptors involved in pathogen identification and absorption, including the mannose receptor, FCRs, scavenger receptors, TLRs, and CRs. They release intracellular granules that kill pathogens locally. However, reactive oxygen species and neutrophil-derived proteinases can damage and impact surrounding tissues. Neutrophils increase their lifespan in an autocrine manner near the site of inflammation (55), and the balance between recruitment and clearance rates determines neutrophil accumulation in inflamed tissues. Apoptosis removes these cells from the tissue, and tissue-resident MQs are captured by phagocytosis. PMNs are well known for eliminating infections as soon as they enter the tissue. Nonetheless, data indicate that neutrophils may directly support adaptive immunity by directing and initiating adaptive immune responses and by attracting immune cells such as T cells and DCs to the site of inflammation. During inflammation, neutrophils can move from the infection site to the adjacent lymph nodes (56), where DCs pick them up after they experience apoptosis. As a result, DCs can convey and present different antigens derived from neutrophils to the T cells. Furthermore, it has been shown that PMNs can acquire antigen-presenting activities (57). Third, as demonstrated in a *Candida albicans* infection model, neutrophils can directly transfer antigens to DCs. DCs cocultured with neutrophils exposed to *Candida albicans* activate specific T cell responses to *Candida albicans* to the same degree as DCs that had directly taken up *Candida albicans* (58).

Many lung disorders are caused by neutrophilic inflammation, which leads to tissue damage. Like MQs and monocytes, DCs regulate neutrophil trafficking and survival at trafficking sites. DCs increase neutrophil infiltration to help remove infection and then control neutrophil recruitment to prevent excessive damage to the infected tissue. In a mouse model, *Propionibacterium acnes* (*P. acnes*) was injected into the skin (intradermally), resulting in a significant increase in neutrophil infiltration at infection sites. The absence of conventional dendritic cells type I (cDC1s) leads to a decrease in pathogenic inflammatory responses due to the reduced infiltration of PMNs and other inflammatory cells (59). Furthermore, lowering cDC1s increases PMN mortality and reduces the ability of neutrophils to produce neutrophil extracellular traps (NETs). This is due to the reduced expression of genes that typically control apoptosis and increased production of pro-apoptotic Bcl-2 proteins.

Furthermore, conventional dendritic cell type I-derived vascular endothelial growth factor (VEGF) has been demonstrated to influence PMN trafficking following infection with *E. coli* in both mice and humans (59). However, through CLEC9A, cDC1s can prevent neutrophil recruitment. To detect tissue damage, CLEC9A binds to F-actin, produced by necrotic cells. In a mouse model of acute pancreatitis, a deficit in CLEC9A raised the rates of morbidity and mortality. A lack of CLEC9A increased neutrophil infiltration, highlighting the significance of CLEC9A in limiting neutrophil infiltration (60). Smoking can also lead to the activation of DCs, which can lead to an increase in the population of NCR-ILC3 cells in the airways and induce chronic inflammation in smokers (61). Another study showed that the activation of DCs by CD40-L can increase their ability to differentiate CD4^+^ T cells into Th17 cells. Finally, these T cells led to lung injury in the sepsis model through the cGAS-STING-dependent pathway (62). In addition, it has been confirmed that in COPD, a decrease in tolerogenic DCs expressing PD-L1 is associated with disease exacerbation, leading to an increase in the population of inflammatory T cells, including Th1 and Th17 cells (63).

*Mycobacterium tuberculosis* (Mtb) is the infectious agent that causes tuberculosis (TB). In 2020, approximately 10 million individuals developed tuberculosis, and 1.5 million died as a result of the disease, making tuberculosis the most significant cause of mortality other than COVID-19 in comparison with other infections. TB typically affects the lungs, where it interacts with many cell types, notably alveolar MQs and PMNs, the primary phagocytes that house the bacteria. The interaction between Mtb and MQs and other immune cells leads to pulmonary inflammatory processes that cause the recruitment of neutrophils, monocytes, B cells, and primed T cells to the lungs. This ultimately culminates in the development of a granuloma (64). Granuloma formation plays a dual role in the containment and persistence of bacteria. This phenomenon helps the host by limiting the spread of Mtb in the body, but it also supports bacterial reproduction and survival, leading to a latent tuberculosis infection. This infection can persist for many years in the host without developing into active TB disease (65). Neutrophils in Mtb infection play two roles; they are believed to protect against infection in the early stages but become more harmful in the later stages of the disease when an increase in neutrophils is linked to lung damage (66, 67).

Moreover, neutrophils can act as a “Trojan horse” that carries Mtb without intracellular death, which can result in systemic infection (68). In chronic tuberculosis infection, neutrophilia is caused by uncontrolled, pathogenic inflammation and malfunctioning neutrophils with decreased antibacterial action that attract and activate additional proinflammatory PMNs (68). A study revealed that IL-17 affects matrix metalloproteinase (MMP) production in lung biopsies from human pulmonary tuberculosis patients and in bronchoalveolar lavage (BAL) and human airway epithelial cells (69). Furthermore, in Mtb-infected mice, repeated BCG vaccination raised the levels of TNF-α, IL-17, MIP-2, and IL-6, all of which aided in the promotion of airway injury. Further evidence has shown that interactions between Th17 cells and neutrophils are beneficial during acute infection and harmful during chronic disease (70). Th17 cell-mediated neutrophil trafficking to the infection site can cause excessive tissue damage from redundant neutrophils, severe inflammation, a poor prognosis, and lung pathology (71).

The results of new studies have revealed the role of NETs produced by neutrophils in the pathogenesis of lung disease (72). The study conducted by Orestis Katsoulis et al. in 2024 showed that rhinovirus infection stimulates the production of NETs by neutrophils, and these traps contribute to disease pathogenesis. NET production through the release of dsDNA into the tissue environment leads to the stimulation of chronic inflammation and lung tissue damage (73). Furthermore, inhibition of NET formation or removal of dsDNA prevents pathological reactions in COPD.

During viral infections, glucose is extensively catabolized by neutrophils. For example, neutrophils isolated from subjects experiencing SARS-CoV-2-induced pneumonia exhibit damaged mitochondria, glycogen build-up in the cytoplasm, and elevated glycogenolysis and glycolysis (74). Further investigation of changes in neutrophil metabolism in response to SARS-CoV-2 may be necessary to enable future COVID-19 treatments. For instance, administering dexamethasone, a glucocorticoid used to treat inflammatory conditions, to individuals with severe COVID-19 significantly lowers mortality. Dexamethasone, in particular, inhibits the production of IFN-stimulated genes (ISGs) while increasing the number of immunosuppressive PMNs. The ability of a drug to reduce harmful neutrophil activity contributes significantly to its success in treating COVID-19 (75). **Table 1** summarizes the role of DCs and neutrophils in the pathogenesis of different lung diseases. Therefore, neutrophils generally play an important role in the pathogenesis of lung diseases through the production of NETs and inflammatory mediators (80).

**Table 1:** The role of dendritic cells and neutrophils in the pathogenesis of lung disease in specific studies.

Disease Type	Indication	Description	Results	Ref.
Severe COVID-19 pneumonia	High blood proportion of degranulated neutrophils	1. Neutrophils display dysfunctional mitochondria 2. Defective oxidative burst 3. ↑ Glycolysis 5. ↑ Glycogen accumulation in the cytoplasm 6. ↑ Glycogenolysis	1. ↑ Formation of NETs 2. ↑ Neutrophil inflammatory response	(74)
LPS-induced acute respiratory distress syndrome	Aggregation and maturation of pulmonary cDCs	1. Pulmonary cDCs regulated neutrophil infiltration 2. Pulmonary cDCs regulate Th1/Th2 response balance	1. ↑ Myeloperoxidase activity in neutrophil 2. ↑ T−bet expression	(76)
Chronic asthma	High levels of neutrophils	1. GM-CSF controls lung DC function 2. T cell-dependent recruitment of neutrophils to the airways.	GM-CSF potentially regulates the DC-T-cell-neutrophil axis	(77)
*S. pneumoniae* pneumonia	DCs are the main APC in the lung	Neutrophils-mediated guide of iNKT cells into the interstitial space via CCL17	1. ↑ Neutrophils extravasation 2. ↑ iNKT extravasation	(78)
Atopic asthma	pDCs provide intrinsic protection against inflammatory responses to harmless antigens	The absence of neutrophils did not have a predominant effect on T cell priming	pDC regulates Th2 cell cytokine production, airway eosinophilia, and goblet cell hyperplasia	(79)

“↑”, increase.

### 2.3 Innate lymphoid cells

Innate lymphoid cells (ILCs) are cells of lymphoid origin divided into three subtypes, namely, ILC1, ILC2, and ILC3, and their functions are similar to those of Th1, Th2, and Th17 cells, respectively (81). These cells are essential in the initial response against pathogens (82). Although natural killer (NK) cells were previously classified in the ILC1 category, they are currently considered a separate subtype that has functions similar to those of cytotoxic T lymphocytes (CTLs) (83, 84). ILCs and NK cells constitute a small part of the immune system cell population in the lung (85). Nonetheless, these cells appear to be crucial to the etiology of long-term lung disease. COPD and asthma are among the diseases in which ILCs may play a role in their pathogenesis (86, 87).

Recent studies have shown that the population of NK cells and ILC1s increases in the peripheral blood of COPD patients (88). Additionally, the proportion of ILC3s is associated with an increase in these patients and has a direct relationship with the severity of the disease (89). Cigarette smoke increases the population of ILC1s in the parenchyma of lung tissue (88). The production of IFN-γ by NK cells and ILC1s leads to increased activation of alveolar macrophages (AMQs) and the release of ROS (90, 91). During this process, activated macrophages secrete different proteases (MMPs and cathepsins), and the destruction of the lung parenchyma leads to pulmonary emphysema (92). Additionally, NK cells can directly and through their cytotoxic functions, which are mediated by perforin and granzyme, promote apoptosis of lung-related cells and contribute to the development of pulmonary emphysema (92). Additionally, ILC3s accelerate the development of COPD by producing inflammatory cytokines such as IL-23, IL-22, and IL-17 (93). It appears that IL-17 can destroy alveoli by increasing the maturation, recruitment, and activity of neutrophils (release of proteolytic enzymes) (94).

ILC2s also play an essential role in the pathogenesis of lung-related viral infectious diseases (95). Additionally, the role of these cells in some lung diseases, such as asthma, is known (96, 97). ILC2s induce Th2 responses by producing IL-13, TSLPs, and IL33 (98). Therefore, in addition to being able to induce the production of IgE from B cells and allergic responses related to IgE in lung tissue, IgE may increase susceptibility and decrease effective immune responses against viral infections by increasing Th2 and its responses (99, 100). Arginase (Arg1) produced by ILC2s is essential in the pathogenesis of lung disease, including COPD and IPF (101). It has also been shown that the inhibition of Arg1 associated with ILC2s may help to improve these diseases. Therefore, Mitolan reported that ILC2s play a central role in the pathogenesis of COPD and IPF (101, 102). The production of TGF-β by ILC2s can also affect the ability of these cells to affect the pathogenesis of IPF (103).

Regarding the role of ILC2s in the pathogenesis of COVID-19, it has been shown that these cells accumulate in lung tissue along with IL-13, and their increase in lung tissue is associated with disease severity (104). Therefore, it is likely that ILC2s play critical roles in development and tissue repair early in life and during severe virus-induced epithelial cell injury, although they may also give signals that promote inflammation and pathology, such as IL-13. This may be due to the differential synthesis of amphiregulin (AREG) (95).

### 2.4 T cells

Disturbances in the regulation of T-cell function can damage different tissues. In many autoimmune diseases, the leading cause of tissue damage is the lack of regulation and homeostasis of T cell responses. T cells are composed of other subtypes, each with specific functions. Various studies have shown that an imbalance in the proportions of different populations of T cells can ultimately worsen conditions in lung-related diseases.

One of the primary immune branches that may play a role in the pathogenesis of lung disease is the Th17/Treg axis, which is associated with an increase in Th17 and a decrease in Tregs. These cells can transform into each other due to their high plasticity. This imbalance is observed in diseases such as COPD, ARDS, sarcoidosis, pulmonary infectious diseases, and even asthma. Th17 cell-produced cytokines, such as IL-17A, are significantly increased in COPD patients. This cytokine significantly impacts the pathogenesis of this disease by affecting naive T cells, lung epithelial cells, and goblet cells.

Analysis of the isolated T cells from severe COVID-19 patients revealed that these cells are overactivated. T cells isolated from these patients express high levels of immune system stimulatory markers, including CD25. However, these cells suppress the expression of the Foxp3 transcription factor, which is a marker associated with Tregs. It has also been shown that T cells isolated from COVID-19 patients have multimodal functions with Th1 and Th2 characteristics (105). These cells can decrease the Treg population, leading to an increase in the production of inflammatory cytokines and, ultimately, a cytokine storm (CS). In addition, the amount of secretory CD25 (sCD25), a marker of excessive stimulation and proliferation of T cells, is significantly increased in patient serum (105).

## 3 MSCs and immune cell communication in lung-related disease

MSCs exert therapeutic effects on diseases through various mechanisms (106). These cells have advantageous properties, such as differentiating into several tissue cell types when various growth stimuli are present (107). Additionally, these cells have approved immunomodulatory properties (108) and can prevent pathological inflammation in inflammatory environments (27, 109). In many cases, the pathological function of immune cells is due to defects in the mitochondria of these cells (108, 110). MSCs use different mechanisms to affect immune cells in pulmonary disease. One of the main mechanisms is the production of exosomes from these cells. Exosomes and extracellular vehicles (EVs) derived from MSCs have a high immunomodulatory potential and can stimulate tissue regeneration and repair (111).

MSCs can regulate immune cell function through the direct or indirect transfer of healthy mitochondria via EVs (31, 112). In addition, different studies have indicated the presence of various immune checkpoint-related molecules that can prevent pathological inflammation and the excessive function of immune cells, including T cells (113). Considering the importance of immune cells in the pathogenesis of lung-related diseases, MSCs have been used for treatment in many studies. Soluble factors produced by MSCs can also affect immune system responses. PGE2, TGF-β, IDO, TSG6, CCL2, and CXCL12 are among these factors (114–116). MSCs can be used in both intact and engineered forms to treat pulmonary disease (117). Engineered MSCs can express different chemokine receptors that increase their migration to the lung site (118). Furthermore, their characteristics can be changed to increase their production of anti-inflammatory factors.

Due to the limitations of using MSCs, including migration to unwanted locations, tumorigenicity potential, allogeneic characteristics, long and complex processes to produce cells of appropriate quality, and painful injection methods, some researchers have resorted to using vesicles derived from MSCs (119). As one of the EVs produced by MSCs, exosomes are small and can pass through small vessels. These vesicles have immunocompatible and biocompatible features and are well tolerated after injection. Their production costs less than cells, and they have not been observed to have tumorigenic properties (120). In the following section, we will discuss the effects of direct communication between MSCs and immune cells in the lung environment and the effect of their vesicles on the responses of resident lung immune cells.

### 3.1 MSC and MQ communication

**Figure 1:**
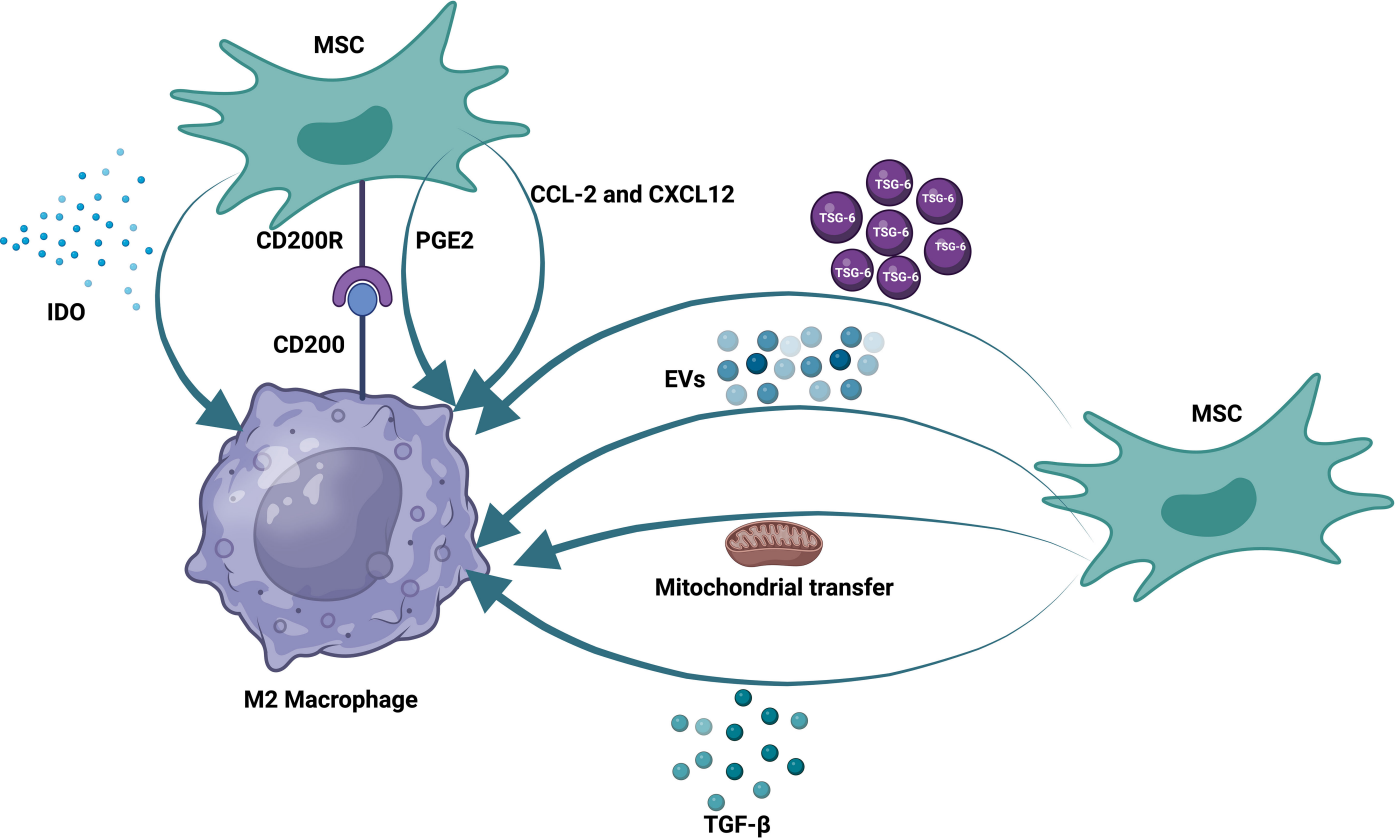
Therapeutic effects of mesenchymal stem cells on macrophages. MSCs communicate with pulmonary macrophages through different mechanisms. These methods include the production of soluble factors, the production of extracellular vesicles, and the transfer of mitochondria. MSCs also express receptors that can directly bind to their ligands on the surface of alveolar macrophages.

Prior research has demonstrated that MSCs can control B, T, and DC functions (121, 122). Moreover, how mesenchymal stem cells and innate immune cells interact is becoming increasingly understood. MSCs can influence macrophage polarization, phagocytosis, and metabolism. As previously noted, the reparative function of MSCs and the MSC-MQ communication makes it possible to employ them for both acute and chronic lung injury (123, 124). In numerous models of chronic lung injury, such as allergy, ragweed, or ovalbumin-induced asthma, the use of MSCs or their release factors and EVs may be helpful (125, 126). MSCs produced TGF-β in asthmatic mice, leading to an increase in regulatory MQs and T-cell expansion. This restored cytokine homeostasis and prevented detrimental allergic responses (127). MSC-EVs alleviated lung damage in bronchopulmonary dysplasia (BPD) model mice by enhancing M2-like interstitial/alveolar MQ polarization and their anti-inflammatory and antiproliferative effects (128). To promote tissue repair in COPD, MSCs reduce the recruitment of neutrophils and MQs into the airways, which decreases IL-1β and IL-6 production (129) (**Figure 1**).

In contrast, MSCs increase growth factors [epidermal growth factor (EGF), VEGF, TGF-β, and hepatocyte growth factor (HGF)] and IL-10 to a greater extent (130). MSCs or MSC-derived extracellular vesicles may mitigate lung fibrosis in models of IPF through their ability to act on MQs, encouraging the proliferation of ATII cells while suppressing the proliferation of lung fibroblasts (131, 132). By modifying pulmonary MQ morphologies and transforming them into an immunoregulatory and anti-inflammatory phenotype, MSC-derived EVs also effectively prevented or reversed lung fibrosis in mice treated with bleomycin (133, 134). Exosomes derived from MSCs can also suppress pyroptosis in alveolar macrophages and inhibit harmful inflammatory responses in ALI (135).

Further research, such as clinical investigations, is required on the underlying mechanisms of MSC activity and the MSC-MQ interaction, especially in chronic lung disorders. Mechanical, large cohort randomized controlled trials are necessary. To date, clinical trials on asthma, COPD, IPF, and silicosis have primarily evaluated the safety of single or double doses of MSC therapy, with a primary focus on the immediate effects of the treatment (136).

Although these trials had positive results, particular attention must be given to the potential of MSC treatment in the long-term stages of IPF to decrease lung function and fibrosis (137). Clinical trials are currently testing MSCs for a variety of conditions, including pulmonary arterial hypertension, liver cirrhosis, aplastic anemia, sepsis, COVID-19 pneumonia, ARDS, pulmonary emphysema, and cancer. Studies on patients with COVID-19-related ARDS and pulmonary emphysema have shown that allogeneic MSCs can be safely administered in specific therapeutic settings. MSCs or MSC-EVs have been used in these clinical investigations to treat lung injury, most commonly to treat ARDS, including after SARS-CoV-2 infection (**Table 2**) (148, 152, 153).

**Table 2:** Macrophage and mesenchymal stem cell communication through different mediators.

No	Category	Mediator Type	Effect on macrophage	Ref.
1	Chemokines	CCL-2, CCL-5, CCL-7, CXCL8, CXCL9, CXCL10, CXCL11, CXCL12	1. ↑ M2 macrophage differentiation 2. ↑ M2 macrophage functions 3. ↑ STAT 3, STAT 6, and PPARγδ transcription factors expression	(138–140)
2	Growth factors	KGF, Ang-1, VEGF, HGF, IGF-1	1. ↓ M1 macrophage differentiation 2. ↓ M1 macrophage functions and cytokine production	(140–142)
3	Inflammatory mediators	NO, TGF-β, COX-2/PGE2, LXA4	1. ↓ Heme oxygenase-1 (HO-1) expression 2. ↑ Macrophage antioxidant activity	(143–146)
4	Anti-inflammatory mediators	IDO, TSG-6, Rv-D1, Rv-E1, E2, Protectins	1. ↑ IL-10 and TGF-β production 2. ↑ Ang-1 functions	(27, 147, 148)
5	Extracellular vesicles	Exosomes, microvesicles, apoptotic bodies	1. ↑ Anti-inflammatory functions 2. ↑ M2 macrophage differentiation 3. ↑ Tissue regenerative properties	(149–151)
6	Mitochondria	Hole mitochondria, mitochondria subunits, and mitochondria transfer by EVs	1. ↑ M2 macrophage polarization 2. ↓ Foam cell formation 3. ↑ Tissue regeneration	(31)

“↑”, increase; “↓”, decrease.

MSCs and MSC-EVs have also been linked to antiviral activities, and their effects have already been described. Due to their anti-inflammatory and tissue regenerative properties, MSCs can potentially treat influenza-induced viral ARDS and pneumonia, partly because of their interaction with MQs. They can also decrease the replication of viruses and shedding by utilizing IDO and LL37 production (154). The angiotensin-converting enzyme 2 (ACE2) receptor, which is widely expressed, allows SARS-CoV-2 to enter cells (155). MSCs lack ACE2 so they are immunosuppressive against SARS-CoV-2 infection and continue to function this way (156). This crucial characteristic supports the therapeutic use of MSCs in COVID-19 (157). During SARS-CoV-2 infection, the interaction between innate immune cells, particularly MQs, and chemicals produced by MSCs and MQs, as previously mentioned, may reduce immunological hyperactivation. MSCs also show lung repair and antifibrotic properties (158). It is thus possible to employ MSCs or MSC-derived EVs as supportive and curative therapies for COVID-19, particularly in severely injured patients (159).

Only limited preliminary statistics are available, as the majority of the trials are still in progress (159). The U.S. ClinicalTrials.gov website has shown encouraging results: patients with COVID-19 treated with MSCs or MSC-EVs have not experienced any side effects, proinflammatory cytokine expression has decreased, and recovery time has improved (148). Studies conducted by non-U.S. governmental websites have demonstrated a favorable correlation between increased IL-10 expression and better lung function outcomes, lower mortality, and beneficial effects on chest imaging results, in addition to enhanced saturation of oxygen in the MSC-treated COVID-19 population (**Table 2**) (157, 158).

### 3.2 MSC communication with DCs and neutrophils

Traditional medication intervention does not substantially affect damaged airways, pulmonary epithelial cells, or other respiratory system pathologies produced by the inflammatory response. However, stem cells have demonstrated significant promise as therapeutics due to their ability to replicate and differentiate in multiple directions, and their immunoregulatory effects (160). The qualities of MSCs make them suitable for treating a wide range of disorders, including common inflammatory diseases of the pulmonary system. Immunological compatibility, for instance, enables MSC transplantation over histocompatibility boundaries, which infrequently trigger an immune reaction (161). Moreover, like endogenous MSCs, exogenous MSCs can migrate to injured tissues through the SDF-1-CXCR4 axis, in which MSCs’ CXCR4 interacts with SDF-1, produced during lung tissue destruction. MSCs can develop into ATII-like cells by activating canonical and non-canonical Wnt pathways, boosting lung tissue regeneration (162). However, the potential for MSC differentiation has not been thoroughly investigated. The ideal culture conditions must be found to enable MSCs to differentiate in the right direction because, under some experimental circumstances, they may also differentiate into myofibroblasts and exacerbate pulmonary fibrosis. Furthermore, the MSC secretome plays a significant role in immunomodulation and tissue regeneration, which is thought to be the primary mechanism by which MSCs can mediate lung damage (125).

Chemokines generated by injured lung tissue can recruit immune cells such as eosinophils, macrophages, neutrophils, and T lymphocytes, among others, to contribute to pulmonary inflammation. All immune cell functions that are involved in the pathophysiology of inflammatory pulmonary disorders, such as neutrophils, effector and regulatory T cells, professional antigen-presenting cells (DCs, B lymphocytes, and MQs), and neutrophils, can be modulated by MSCs. MSCs modulate immunological responses via the juxtacrine or paracrine pathways (163). MSCs can modulate innate immune cells and thus have indirect regulatory effects on adaptive immune cells. MSCs, for example, can convert mature DCs into a suppressive immature phenotype and increase IL-10-producing plasmacytoid DC (pDC) development, resulting in effector T-cell suppression and Treg production. MSCs release anti-inflammatory chemicals, including IDO, PD-L1, and IL-1ra, directly suppressing effector T cells (164). In addition to directly suppressing effector T cells, MSCs can decrease the production of Th17, Th2, and Th1 cells by altering the antigen-presenting activity of DCs in an IL-10, IL-6, and PGE2-dependent manner (165). Following contact with MSCs, DCs become immature, with diminished antigen presentation capacity due to decreased expression of the costimulatory molecules (CD80 and CD86) and MHC I and II. Moreover, MSCs can induce a tolerogenic phenotype in DCs and encourage the polarization of M1 macrophages, which are inflammatory, into M2 macrophages, which are immunosuppressive. MSCs suppress the production of inflammatory cytokines (IL-1β, IL-12, and TNF-α) in macrophages and DCs while promoting the production of anti-inflammatory cytokines (TGF-β and IL-10), leading to improved tissue repair and regeneration (165). M2 macrophages and rDCs induce MSCs to produce the immunosuppressive HLA-G5, which encourages the development and multiplication of Tregs (166). Neutrophils play an essential part in acute inflammation. MSCs can interact with neutrophils to increase neutrophil recruitment (started by MIF and IL-8) and decrease the apoptosis of resting neutrophils (initiated by IL-6) (167). Moreover, the reduction of uncontrolled inflammation in a murine vasculitis model mediated by superoxide dismutase (SOD) suggests that MSCs have anti-inflammatory properties that lessen tissue damage. It has been discovered that MSC-EVs restrict neutrophil migration into the pulmonary parenchyma of mice suffering from acute lung injury caused by *E. coli* endotoxin (168).

However, MSCs do not always suppress the immune system and may exhibit pro-inflammatory features in specific conditions. For example, MSCs can increase monocyte, macrophage, and neutrophil infiltration into tumors via chemokines. Recent research on ecto-ATP synthase has emphasized the relevance of this cell-surface expressed protein in numerous disorders (**Table 3**) (169). Determining invasion, cell proliferation, and chemoresistance in tumor tissue is contingent upon the extracellular ATP (eATP) concentration. Recent cancer research has suggested that P2XRs function as specific eATP receptors on the plasma membrane. Lung cancer cells’ P2X7Rs are activated by this extracellular ATP, which then triggers the phospho-ERK1/2/c-Fos axis. MSCs play a role in the development of lung cancer by promoting tumor growth and migration (169). One of the most prevalent chronic lung diseases is COPD. MSCs have been proven in trials to reduce emphysema and inflammatory symptoms and have a positive therapeutic impact on experimental COPD. Mesenchymal stem cells from various sources can decrease the average linear intercept, cell death, and neutrophil infiltration (170). Systemic administration of MSCs in an LPS-induced ARDS mouse model dramatically reduced alveolar damage and inflammation. MSCs reduced neutrophil influx and TNF-α generation in immune cells inserted into the lungs through paracrine IL-10-dependent mechanisms (171). Since the COVID-19 pandemic began, multiple clinical trials examining MSC therapy have been conducted, with encouraging findings for the treatment of ARDS. The number of DCs in lung tissue rises during ARDS brought on by hemorrhagic shock or LPS, and the maturation of pulmonary DCs enhances lung inflammation and pathological damage (172). Hound-derived AD-MSCs and mouse-isolated BM-MSCs can turn mature DCs into rDCs, suppressing other DC activation and inhibiting inflammatory cytokine release *in vitro*. Mechanistically, paracrine HGF, released from human UC-MSCs and mouse BM-MSCs, may cause the AKT signaling pathway to activate, driving mature DC differentiation into rDCs and inhibiting T lymphocyte proliferation to mitigate lung damage in mouse models (173). MSCs decrease the number of DCs and M1 macrophages, inhibiting antigen presentation (174). Su et al. reported that an MSC-derived supernatant caused neutrophil death in lung tissue after LPS-induced acute lung damage. MSCs can boost neutrophil phagocytic activity and germ clearance (175).

**Table 3:** MSC therapy in randomized controlled trials in patients with severe acute respiratory distress syndrome (ARDS), particularly COVID-19-related ARDS.

Phase	Patient Cohort and Size	Intervention	Outcomes and Results	Reference/Trial Number
Phase 1	Moderate to severe ARDS, 12 patients.	Allogeneic adipose-derived MSCs were administered once intravenously at 1 million cells/kg or a placebo.	No toxicity or serious adverse events (SAEs) were observed. Hospital stay, ventilator-free days, and biomarker levels were unchanged.	NCT01902082
Phase 1 (STAR)	Moderate to severe ARDS, 9 patients.	Allogeneic bone marrow-derived MSCs were administered once intravenously at doses of 1, 5, or 10 million cells/kg, with 3 patients per dose.	Safety confirmed; allogeneic bone marrow-derived MSCs were well tolerated in a single dose.	NCT01775774
Phase 2a (STAR)	Moderate to severe ARDS, 60 ventilated patients.	Allogeneic bone marrow-derived MSCs were administered once intravenously at a 2:1 ratio of either 10 million cells/kg or placebo.	There was no significant difference in 28-day mortality after adjusting for APACHE III scores.	NCT02097641
Nested within Phase 2a (STAR)	Moderate to severe ARDS, 27 patients.	Allogeneic bone marrow-derived MSCs were administered once intravenously: 10 million cells/kg for 17 patients and placebo for 10 patients.	Significant reductions in airspace total protein, Ang-2, IL-6, and soluble TNF receptor-1 concentrations after MSC treatment.	NCT02097641
Phase 1/2a	COVID-19-induced ARDS, 24 patients.	Umbilical cord-derived MSCs were administered twice intravenously (100 million cells per infusion) with heparin; the control group received a placebo with heparin.	No adverse or serious adverse events; noted improvement in patient survival and recovery time.	NCT04355728
Phase 1 (REALIST)	COVID-19 with mild to severe ARDS, 9 patients.	Umbilical cord-derived MSCs (CD362 enriched) were administered once intravenously in doses of 100, 200, or 400 million cells per infusion, with 3 patients per dose.	Treatment was well tolerated, with no dose-limiting toxicity observed, supporting progression to Phase 2 trials.	NCT03042143
Phase 1/2a	Critically ill COVID-19 patients, 40 patients.	Umbilical cord-derived MSCs combined with standard care (oseltamivir and azithromycin) were administered once intravenously at 1 million cells/kg or placebo with standard care.	Improved survival rates were observed with no significant changes in ICU stay or ventilator use; a reduction in IL-6 levels was noted.	NCT04457609
Phase 2	Severe ARDS in COVID-19, 100 patients.	Umbilical cord-derived MSCs were administered three times intravenously (40 million cells per infusion) or placebo.	Improved whole-lung lesion volume; no difference in serious adverse events reported.	NCT04288102
Phase 2b	Mild to severe ARDS in COVID-19, 45 patients.	Umbilical cord-derived MSCs were administered three times intravenously at 1 million cells/kg in 21 patients or placebo in 24 patients.	No serious adverse events linked to repeated cell infusions; no significant changes in the PaO2/FiO2 ratio between groups.	NCT04333368

**Figure 2:**
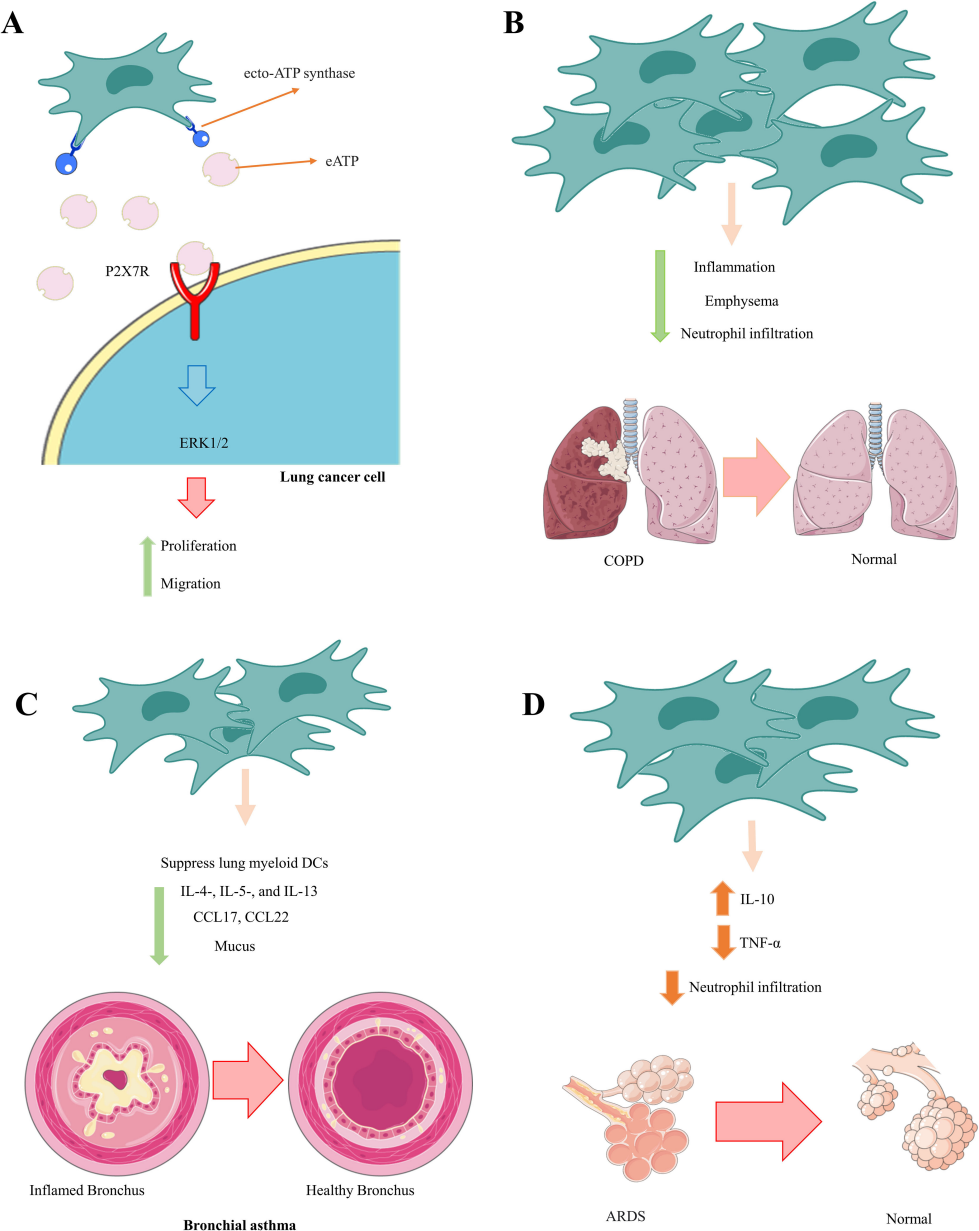
Therapeutic effects of MSCs in communication with neutrophils. MSCs play an essential role in preventing the pathological and damaging functions of neutrophils by inhibiting the calling of neutrophils and reducing their inflammatory function. **(A)** Lung cancer, **(B)** COPD, **(C)** Bronchial asthma, **(D)** ADRS.

Zeng et al. established that the positive benefits of MSCs in a mouse model of bronchial asthma were due to MSC-mediated inhibition of lung myeloid DC functions (176). MSC-treated asthmatic mice produced immature DCs with reduced antigen presentation and naive T-cell activation. Furthermore, DCs from mice that received MSCs could not move adequately to regional lymph nodes and create an adequate quantity of CCL17 and CCL22, which are critical for migrating effector Th2 cells in inflamed lungs. As a result, MSC-treated asthmatic mice had lower numbers of IL-4-, IL-5-, and IL-13-producing Th2 cells; lower blood IgE levels; fewer lung-infiltrated eosinophils; and less mucus formation. These MSC-mediated actions resulted in considerable attenuation of pulmonary inflammation, elimination of bronchial hyperresponsiveness, and significantly improved lung function in MSC-treated asthmatic mice (176). Furthermore, BM-MSCs significantly reduced serum IgE and IgG1 levels, the number of goblet cells, and the number of neutrophils and eosinophils in BAL fluid in severe asthma models induced by ragweed or toluene diisocyanate. They also suppressed aryl hydrocarbon receptor (AHR) and collagen deposition (177) (**Figure 2**). Future research could focus on increasing the homing of MSCs to injured lung tissue.

### 3.3 MSC communication with T cells

Inflammation and immunological dysregulation are prevalent characteristics of infectious and non-infectious pulmonary disorders, such as COPD, where CTLs and neutrophils have a crucial role in injury, and asthma, where eosinophils and Th2 cells are more prominent (178, 179). AMQs resident in the lung are essential for improving fibrosis in restrictive diseases such as IPF (180). The chronic and acute lung disease observed in these conditions is consistently associated with abnormal immune responses and fibrosis. In COPD, inflammation resulting from AMQs, CTLs, and neutrophils results in progressive airflow limitation, small airway fibrosis, and alveolar damage. Conversely, asthma involves mast cells, eosinophils, and Th2 lymphocytes, contributing to increased airway hyperresponsiveness and bronchoconstriction (181, 182).

MSCs are commonly used to impede CD8^+^ and CD4^+^ T lymphocyte activity (183). This suppression occurs during the G0 phase of the cell cycle, inhibiting T-cell proliferation (184). MSCs play a role in modulating immune responses and lymphocyte proliferation by inducing the production of Tregs through two mechanisms. First, MSCs engage in direct cell-to-cell interaction with CD4^+^ T lymphocytes. Second, they secrete prostaglandin E2 (PGE2) and TGF-β1 (184). MSCs can increase the Treg population by inhibiting the differentiation of CD4^+^ T lymphocytes into pro-inflammatory cells, including Th1 and Th17. Numerous studies have demonstrated the therapeutic effects of MSC injection in combination with Th17 suspension in mouse models of autoimmune disease. In the early stages of the disease, there is an increase in the levels of lymphocytes, specifically CD4^+^ CD25^+^ Foxp3^+^ cells (121). MSCs and EVs derived from these cells can successfully treat COVID-19. The research findings by Motallebnezhad et al. revealed a growth in the proportion of Tregs in peripheral blood mononuclear cells (PBMCs) exposed to exosomes derived from MSCs of COVID-19 patients  (185).Communication between MSCs and preactivated T cells was found to have a significant impact on the CCR2-CCL2 axis. This interaction results in the suppression of T-cell function, decreased T-cell infiltration into lung tissue, and alleviated lung injury (186). Chemokines and their receptors are believed to play a crucial role in developing this complication (187). CCL2, also known as MCP-1, is a leading chemoattractant for various cell types, with a powerful attraction to T cells. In a mouse model, the injection of MSCs results in a significant increase in CCL2 levels in the BAL fluid of mice (188).

Additionally, there was an increase in the percentage of T cells in lung tissue expressing CCR2, specifically the CCR2^+^ CD4^+^ T cell subgroup. A reduction in T lymphocyte infiltration, lung pathological damage, and a significant increase in survival accompanied these changes. The inhibitory effects of MSCs were successfully reversed *in vivo* by both CCR2 and CCL2 antagonists. These results imply that the primary mechanism behind MSCs’ suppression of T-cell immunity may be the generation of CCR2^+^ CD4^+^ T cells. The adoptive transfer of CD4^+^CCR2^+^ T cells mitigates the development of inflammation and fibrosis (189).

Interestingly, a more significant proportion of the FOXP3^+^ subset was observed within the CCR2^+^ CD4^+^ T cell population. These findings suggest that CD4+ CCR2+ T cells can regulate immune responses and play a crucial role in suppressing lung inflammation and fibrosis (189). Notably, when exposed to an inflammatory environment in the lung, MSCs secrete a significant quantity of CCL2, which attracts CCR2-expressing T cells to recruit to the lung tissue. Additionally, the proportion of CD4^+^CCR2^+^ T lymphocytes in MSCs remained consistently more significant than that in mice treated with IPS. This sustained increase in CD4^+^CCR2^+^ T lymphocytes may be attributed to the high concentration of CCL2 produced by MSCs in the early stages and the subsequent activation of lung interstitial cells (190, 191). Due to the immunoregulatory effects of CD4^+^CCR2^+^ T lymphocytes, mice receiving MSC prophylaxis exhibited improved clinical symptoms, pathological manifestations, and survival rates compared to mice in the IPS group. These results offer a theoretical foundation for using MSCs, CCL2, or CD4^+^CCR2^+^ T cells to treat immune-related diseases (192).

Individuals with COPD exhibit an imbalance in the lymphocyte subpopulations, characterized by increased CD4^+^ and CD8^+^ T lymphocytes and decreased Tregs. Specifically, CD8^+^ T lymphocytes have been implicated in the apoptotic mechanisms that lead to lung tissue destruction induced by TNF-α. In an emphysema model, the BAL fluid contained fewer CD4^+^ T cells due to administering either one or two doses of MSCs. However, only the two-dose regimen decreased the proportion of total lymphocytes (193). Additionally, total thymic cell count and weight increased in emphysematous animals; this could be explained by the migration of undifferentiated lymphocytes to the thymus, where they develop into T cells. There was a reduction in thymus weight, total cell count, and CD8^+^ T lymphocyte count only with the two-dose MSC treatment (194).

Additionally, administering two doses of MSCs decreased CD8^+^ and CD4^+^ T lymphocyte counts in the cervical lymph nodes (cLNs). MSCs may cause immunosuppression in lymphoid tissues by stopping T cells that are still immature in the G0/G1 cell cycle phase, thereby not affecting the cells already activated in inflammatory foci (195). An MSC infusion has been shown in numerous rodent studies of COPD to reduce inflammation and parenchymal damage; some reports have also implicated MSC paracrine factors, including keratinocyte growth factor (KGF), HGF, vascular endothelial growth factor, and epidermal growth factor (196–200). In addition, in animal models of elastase-induced emphysema or cigarette smoke-induced COPD, MSC administration has been shown to mitigate lung damage and abnormal inflammation (201, 202). MSCs secrete EGF, which induces the production of secretory leukocyte protease inhibitors (sLPIs). sLPIs act as inhibitors, safeguarding epithelial tissue against degradation caused by serine proteases. When MSCs were infused into a rat model of cigarette smoke-induced lung injury, proinflammatory cytokines such as IL-1β, TNF-α, MCP-1, and IL-6 were downregulated (195). Moreover, MSC treatment led to the upregulation of VEGF and TGF-β. Additionally, MSC therapy can decrease COX-2 and COX-2-mediated PGE2 synthesis in alveolar macrophages, consequently decreasing inflammation (144, 193, 194).

In asthma, MSC treatment via inhalation of cockroach extract, ovalbumin, or toluene diisocyanate has modulated the immune environment in rodent disease models (203, 204). This modulation occurs through the generation of Tregs and the inhibition of Th2 responses (204, 205). As a result, disease symptoms are reversed, accompanied by a decrease in Th2 cytokines such as IL-4, IL-13, and IL-5 and a reduction in IgE levels, MMP deposition, and mucus production (206–208). The upregulation of TGF-β1 and IL-10 in PBMCs by MSC-produced exosomes led to the enhancement of Treg proliferation and immune suppression. Additionally, the findings indicated that the regulation mediated by MSC-derived exosomes involved APCs rather than the CD4^+^ T-cell-dependent pathway. These results shed light on the crucial function of exosomes in MSCs’ immunological regulation and highlight the potential therapeutic applications of MSC-derived exosomes in asthma treatment (209).

**Figure 3:**
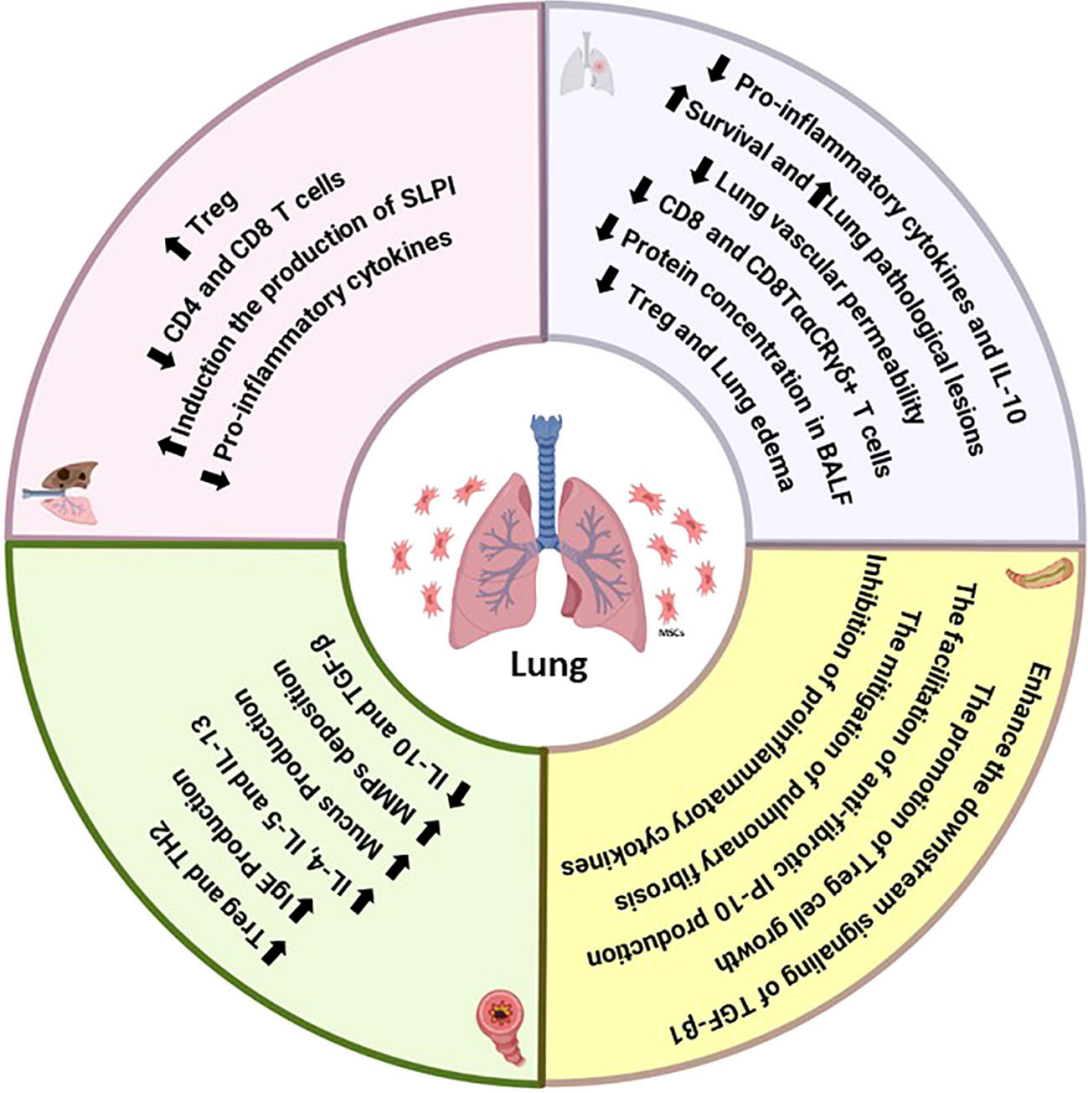
Summary of the immunomodulatory effects of MSCs on the functions of T cells in lung diseases.

During the initial inflammatory phase, MSCs also enhance survival, reduce pulmonary and intestinal inflammation, improve intestinal barrier function, and decrease intestinal recruitment of CD8^+^ T lymphocytes in ALI, regardless of antibiotic treatment (210, 211). Antibiotic-induced reduction of intestinal bacterial communities has been linked to increased lung and gut damage and death, which may worsen the onset or progression of pulmonary disease. In LPS-induced ALI mice, MSCs significantly decreased total protein, survival, inflammatory cells, and lung pathological lesions in BAL fluid. Mice with ALI caused by a gut microbiota imbalance exhibited more severe gut damage and significant mortality, while MSCs mitigated gut damage and enhanced intestinal mucosa barrier function. Furthermore, MSCs reduced the inflammatory response in BAL fluid by decreasing the levels of IL-1β, IFN-γ, IL-1α, IL-6, and TNF-α (212–214). The immunomodulatory effects of MSCs on the pulmonary system involve T-cell differentiation, with therapeutic benefits mediated by reduced recruitment of CD8^+^ Ly6C^+^ T lymphocytes and decreased IFN-γ levels in the LPS/MSC group, potentially preventing tissue injury caused by excessive IFN-γ production by CD8^+^ T cells. The maintenance of host immune homeostasis relies heavily on the gastrointestinal mucosal immune system. Effector memory CD8^+^ T cells, found in intraepithelial lymphocytes (IELs), play a crucial role in maintaining the gut mucosal barrier’s integrity. Studies have demonstrated that inhibiting CD8^+^ T lymphocytes can control immunological reactions and reduce lung inflammation-related disease (212, 215, 216). In the context of intestinal mucosa, mesenchymal stem cells have been shown to reduce CD8^+^ T-cell infiltration, thereby ameliorating injury. This observation aligns with changes in the lungs (**Figure 3**).

Consequently, mesenchymal stem cells exhibit an immunosuppressive effect in the lung and stomach during ALI. Additionally, MSCs may facilitate intestinal improvement by modulating the composition of the intestinal bacterial population. The interaction between MSCs and the microbiota influences the immunomodulatory functions of mesenchymal stem cells, including their ability to regulate immune responses (217). Notably, administering antibiotics to ALI mice resulted in the depletion of bacterial communities, exacerbating lung and intestinal damage and immune responses. MSCs, however, decreased the proportion of CD8^+^ CTLs, alleviating inflammatory responses in mice. The percentages of both CD8^+^ T cells and CD8TααCR T γδ^+^ lymphocytes were significantly reduced in the ALI mice treated with MSCs. This implies that MSCs’ anti-inflammatory qualities aid in ALI recovery by influencing the generation of inflammatory cytokines by T cells and MQs (217, 218). ALI mice had an enhancement in anti-inflammatory factors, including IL-10, and a reduction in TNF-α, MIP-2, and IFN-γ levels compared to those in control mice. Transplantation of UC-MSCs improves ALI by restoring the reduced levels of alveolar CD4^+^CD25^+^ Foxp3^+^ Treg cells and maintaining a balance between anti-inflammatory and proinflammatory factors in ALI mice (219, 220). UC-derived MSCs can directly produce or stimulate immune cells to release immunoregulatory cytokines *in vivo* during ALI. Transplantation of MSCs significantly decreases systemic and pulmonary levels of IFN-γ, TNF-α, and MIP-2 while increasing IL-10 levels. Foxp3^+^CD25^+^CD4^+^ Tregs play an essential role as lung-protective immunomodulators in ALI following MSC treatment, acting through IL-10 on several cytokine-dependent and cellular inflammatory targets (171, 221, 222). Natural regulatory (Foxp3^+^CD25^+^CD4^+^) T cells regulate the production of proinflammatory cytokines during infection, with Foxp3^+^CD25^+^CD4^+^ Tregs promoting IL-10 production. The combined increase in the percentage of Foxp3^+^CD25^+^CD4^+^ Tregs and the equilibrium between proinflammatory IFN-γ, MIP-2, TNF-α, and anti-inflammatory IL-10 levels contributed to the therapeutic benefits of MSCs in ALI mice exposed to endotoxin-induced damage (171, 223). Intratracheal transplantation of MSCs in experimental endotoxin-induced ALI resulted in a significant survival effect due to the expansion of Foxp3^+^CD25^+^CD4^+^ Tregs, which reduced pulmonary vascular permeability. This decreased lung edema through the modulation of pro- and anti-inflammatory cytokines. These findings suggest the potential of MSC-based therapy for ALI patients (224–226).

Inhibition of the TGF-β signaling pathway, which is crucial for continuous IL-6/STAT3 gene activation, plays a role in decreasing Treg development in connective tissue disease interstitial pneumonia (CTD-IP) and promoting myofibroblast formation in CTD-UIP HLFs. MSCs can enhance the downstream signaling of TGF-β1, which controls the activation of IL-6/STAT3, leading to the promotion of Treg growth and the facilitation of antifibrotic IFN-gamma-inducible protein of 10 kDa (IP-10) production. Consequently, this process could stop pulmonary fibrosis from worsening in patients with autoimmune diseases (227). Additionally, studies have indicated the crucial involvement of CD8^+^ T cells in initiating pulmonary fibrosis. Human MSCs have been shown to alleviate pulmonary fibrosis and enhance lung function by suppressing bleomycin-induced infiltration of human T cells and the production of proinflammatory cytokines in the lung. Significantly, the mitigation of pulmonary fibrosis by human MSCs is mediated through the PD-1/PD-L1 pathway (113). Additionally, aberrant expression of PD-1 has been observed in circulating T cells and lung tissue of patients with pulmonary fibrosis (**Table 4**) (228).

**Table 4:** The effect of mesenchymal stem cell transplantation on T cell function.

Disease	Pathologic immune mechanism	MSCs therapy outcome
COPD	↑ CD4 and CD8 T cells ↓ Treg ↑ Pro-inflammatory cytokines (TNF-α, IL-1, IL-6, etc)	↓ CD4 and CD8 T cells ↑ Treg ↓ Pro-inflammatory cytokines (TNF-α, IL-1, IL-6, MCP-1) ↑ Induction of the production of secretory leukocyte protease inhibitor (SLPI)
Asthma	↓ Treg ↑ TH2 ↑ IgE production ↑ IL-4, IL-5 and IL-13 ↑ Mucus production	↑ Treg ↓ TH2 ↓ IgE production ↓ IL-4, IL-5 and IL-13 ↓ Mucus production ↓ MMPs deposition ↑ IL-10 and TGF-β
Acute lung injury (ALI)	↑ CD8 T cells ↑ Protein concentration in BALF ↓ Survival ↑ Lung pathological lesions ↓ Treg ↑ Pro-inflammatory cytokines (TNF-α, IL-1, IL-6, etc) ↓ IL-10	↓ CD8 and CD8TααCRγδ+ T cells ↓ Protein concentration in BALF ↑ Survival ↓ Lung pathological lesions ↑ Treg ↓ Pro-inflammatory cytokines (TNF-α, IFN-γ, MIP-2, IL-1, IL-6, etc) production from T ↑ IL-10 by production from T ↓ Lung vascular permeability ↓ Lung edema
Pulmonary fibrosis	↑ IL-6/STAT3 Signaling ↓ Treg ↑ Myofibroblast formation ↑ Aberrant expression of PD-1	↑ Downstream signaling of TGF-β1 ↑ Treg growth ↑ Anti-fibrotic IP-10 production ↓ Pulmonary fibrosis through the PD-1/PD-L1 pathway ↓ Proinflammatory cytokines

“↑”, increase; “↓”, decrease.

## 4 Conclusion and future perspectives

In lung disease, due to the release of chemotactic factors and DAMPs by lung cells, various types of immune cells are recruited to the lung tissue and contribute to lung tissue pathology. Considering the proven therapeutic role of MSCs in treating lung diseases (111, 229, 230), it is essential to investigate the mechanisms underlying this therapeutic potential. MSCs have antifibrotic effects and prevent pulmonary fibrosis. In addition, MSCs can differentiate into different cell types, leading to a reduction in lung damage. Furthermore, their immunomodulatory role is vital for their therapeutic potential. As we have shown before, MSCs play a critical role in regulating the immune system in the liver (33). In this study, we investigated the therapeutic effect of MSCs on the lung immune axis. Studies have shown that MSCs and their EVs can improve lung tissue function in pulmonary diseases by increasing the M2/M1, Treg/Th17, Treg/Th1, and N2/N1 ratios and modulating the Th2 cell response. However, most of these studies used intact MSCs. It is suggested that their therapeutic potential can be increased by developing MSCs with more significant immunomodulatory potential through the CRISPR/CAS9 technique and 3D cultures, as well as by increasing immune checkpoint-related molecule expression (231). Although many studies on the therapeutic application of MSCs in lung diseases have entered clinical trials, no products based on these cells have yet been approved by the FDA, and more studies are needed for this purpose.

There are concerns and limitations regarding the therapeutic use of MSCs in treating various diseases, including pulmonary diseases. If these cells migrate to niches with high proliferative properties (after transplantation into patients) they may significantly increase cancer risk and its progression with immunomodulatory properties, high proliferation, and iatrogenic differentiation. Furthermore, allogenic features should be considered when using these cells in treatment. MSCs express HLA-related molecules on their surface and cannot be used off-the-shelf in the clinic. The cell preparation process must be done personally. For this purpose, byte cells with decreased expression of HLA molecules and other allogeneic molecules on their surface should be produced. However, this may affect their therapeutic potential, and preparing these cells for clinical use is very difficult and requires good manufacturing practice (GMP) conditions to prevent inappropriate differentiation *in vitro* and cell contamination.

## Glossary

ALI: Acute lung injury
MSCs: Mesenchymal stromal/stem cells
EVs: Extracellular vehicles
MSCs-EVs: MSC-derived EVs
TNF-α: Tumor necrosis factor-α
Th: T helper cell
Treg: Regulatory T
TLRs: Toll-like receptors
ARDS: Acute respiratory distress syndrome
LPS: Lipopolysaccharides
AMs: Alveolar macrophages
NF-κB: Nuclear factor kappa B
IL: Interleukin
DCs: Dendritic cells
MQ: Macrophage
DAMPs: Damage-associated molecular pattern molecules
MHC: Major histocompatibility complex
IPF: Idiopathic pulmonary fibrosis
MCP-1/CCL2: Monocyte chemoattractant protein-1
FCRs: Fc receptors
PMNs: Polymorphonuclear cells
VEGF: Vascular endothelial growth factor
COPD: Chronic obstructive pulmonary disease
TGF-β: Transforming growth factor beta
TB: Tuberculosis
Mtb: *Mycobacterium tuberculosis*
MMP: Matrix metalloproteinase
BAL: Bronchoalveolar lavage
ILCs: Innate lymphoid cells
CTL: Cytotoxic T cells
Arg1: Arginase
sCD25: Secretory CD25
CS: Cytokine storm
ACE2: Angiotensin-converting enzyme 2
